# Impact of Serum Biomarkers and Clinical Factors on Intensive Care Unit Mortality and 6-Month Outcome in Relatively Healthy Patients with Severe Pneumonia and Acute Respiratory Distress Syndrome

**DOI:** 10.1155/2014/804654

**Published:** 2014-03-03

**Authors:** Chia-Cheng Tseng, Wen-Feng Fang, Sum-Yee Leung, Hung-Chen Chen, Ya-Chun Chang, Chin-Chou Wang, Huang-Chih Chang, Meng-Chih Lin

**Affiliations:** ^1^Division of Pulmonary and Critical Care Medicine, Department of Internal Medicine, Kaohsiung Chang Gung Memorial Hospital and Chang Gung University College of Medicine, Kaohsiung 83301, Taiwan; ^2^Department of Respiratory Care, Chang Gung University of Science and Technology, Chiayi 813, Taiwan; ^3^Department of Respiratory Therapy, Kaohsiung Chang Gung Memorial Hospital and Chang Gung University College of Medicine, Kaohsiung 83301, Taiwan

## Abstract

*Objectives*. This study aimed to identify the independent biomarkers and clinical factors that could predict ICU mortality and 6-month outcomes in relatively healthy patients with severe pneumonia and acute respiratory distress syndrome (ARDS). *Patients and Methods*. We prospectively enrolled patients with severe pneumonia-related ARDS that required mechanical ventilation. Patients were excluded if they were unable to take care of themselves. Several biomarkers and clinical factors were evaluated prospectively on day 1 and day 3 after ICU admission. All biomarkers and clinical factors were collected for analysis. *Results*. 56 patients were enrolled in this study. We determined that the initial appropriate antibiotics use was an independent clinical factor and day 1 high-mobility group protein B1 (HMGB1) concentration was an independent biomarker for ICU mortality. Interestingly, we also found that a low day 1 albumin level was an independent biomarker for predicting patient life dependence 6 months after a pneumonia event. *Conclusion*. Patients with severe pneumonia and ARDS requiring mechanical ventilation experience high rates of ICU mortality or disability, even if they were quite healthy before. Initial appropriate antibiotics use and day 1 level of HMGB1 were independent factors for predicting ICU mortality. Day 1 albumin level was predictive of 6-month patient life dependence.

## 1. Introduction 

Pneumonia is associated with a high morbidity and mortality rate throughout the world [1]. Acute lung injury (ALI) and acute respiratory distress syndrome (ARDS) are important denouements of severe pneumonia and are associated with high mortality rates [2, 3] despite recent improvement in prognosis [4–6]. Sepsis (both from pulmonary and extrapulmonary origin) is a leading etiology of ALI/ARDS, and lung infection may account for up to 50% of ARDS cases [3, 7].

Although patients with severe pneumonia and ARDS can survive, survivors also frequently experience major long-term morbidities that impair their pulmonary, neuromuscular, physical, cognitive, and psychological function. In turn, these impairments affect the survivors' overall quality of life (QOL) [8, 9]. Previous studies also suggested that long-term survival is unaffected by ARDS status; however, ARDS severely affects QOL, functional independence, and cognitive function [10–12]. In relatively healthy patients bearing few underlying diseases, severe pneumonia with ARDS status rarely occurs; however, patients that are relatively young and healthy when they develop ARDS may never recover completely and may experience ongoing functional limitations after an episode of critical illness [13]. This may be attributed to persistent intensive care unit- (ICU-) acquired weakness, in addition to a variety of other physical and mental health impairments [14]. To date, few studies have been designed to assess the possible factors responsible for disability status after hospital discharge in relatively healthy patients with severe pneumonia and ARDS status. Therefore, this study prospectively evaluated the possible clinical factors and biomarkers of predicting ICU mortality and 6-month outcomes in relatively healthy patients with severe pneumonia and ARDS.

## 2. Patients and Methods 

### 2.1. Setting and Study Design

This prospective study was conducted at Kaohsiung Chang Gung Memorial Hospital, a 2,400-bed tertiary teaching hospital in southern Taiwan. The study was conducted from July 1, 2009, to June 30, 2011, for adult intubated patients (aged ≥18 years) presenting with severe pneumonia and ARDS. Patients known to have tuberculosis or severe immunosuppression, such as those with human immunodeficiency virus or solid-organ or bone marrow transplantation, were excluded from the study. Furthermore, patients were excluded if they had complicated underlying diseases, were unable to take care of themselves, or had a Charlson's comorbidity index (CCI) >5. The study was approved by the Institutional Review Board of Chang Gung Memorial Hospital, and written informed consent was obtained from the patients or their legal representatives.

### 2.2. Definitions

Pneumonia was defined according to modified criteria proposed by the United States Center for Disease Control and Prevention [15]. This definition requires that two of the following criteria be satisfied: fever (increase in body temperature of ≥1°C or body temperature >38.3°C), leukocytosis (25% increase and leukocyte count ≥10,000 mm^3^) or leukopenia (25% decrease and leukocyte count ≤5,000 mm^3^), and purulent tracheal secretions (>25 neutrophils per high-power field). It also requires that one of the following be satisfied: new and persistent infiltrates appearing on the chest radiograph, the same microorganisms are isolated from pleural fluid and tracheal secretions, radiographic cavitation, histological proof of pneumonia, or positive cultures from bronchoalveolar lavage (≥1 × 10^4^ colony-forming units/mL). This study included both community-acquired pneumonia (CAP) and hospital-acquired pneumonia (HAP); we defined HAP as pneumonia that occurred 48 hours after hospital admission [16], and all other cases were classified as CAP. ARDS status was defined as arterial oxygen tension/fraction inspired oxygen (PaO_2_/FiO_2_) < 200 [17].

Antibiotic appropriateness was determined by considering not only antimicrobial susceptibility results [18] but also the results of an evaluation of the initial response to empirical antibiotic treatment for underlying pneumonia. After 7 days of antibiotic treatment, the clinical status of treatment was reevaluated and classified as “appropriate” if the fever had subsided, sputum production had decreased, pneumonia infiltration regression was observed on the chest radiograph, and laboratory data (including white blood cell counts, C-reactive protein levels [CRP], procalcitonin [PCT] levels, PaO_2_/FiO_2_) had improved. Additionally, if antibiotic administration was appropriate, subsequent antimicrobial susceptibility results indicated that the isolated pathogen was sensitive to the antibiotic; otherwise, the treatment was deemed “inappropriate.” If clinical status could not be determined as “appropriate” or “inappropriate,” or if insufficient data were available to permit evaluation of microbiological outcome, the result was designated as “indeterminate.”

Patients who survived after ICU discharge were defined as “ICU survivors” and patients who died during ICU admission were defined as “ICU nonsurvivors.” Patients who survived after hospital discharge were divided into two groups: life dependent and life independent. A patient was categorized as having 6-month life dependence if their Barthel's Index [19] was below or equal to 30 at the 6-month followup after hospital discharge; all others were categorized as having 6-month life independence.

### 2.3. Data Collection and Laboratory Assays

We recorded the age, gender, pneumonia type, initial PaO_2_/FiO_2_ value, and CCI of every included patient. On the seventh day after ICU admission, we evaluated the antibiotic appropriateness according to the definition described above and recorded this as a clinical factor. We also prospectively calculated every patient's disease severity scores, such as the Acute Physiology and Chronic Health Evaluation II (APACHE II) score, Sequential Organ Failure Assessment (SOFA) score, and Simplified Acute Physiology Score II (SAPS II) on days 1 and 3 after ICU admission. In addition, blood samples were evaluated in each patient for several biomarkers, including CRP, PCT, D-dimer, lactate, albumin, high-mobility group protein B1 (HMGB1), interleukin-8 (IL-8), and interleukin-10 (IL-10) on days 1 and 3 after ICU admission.

For evaluating CRP, PCT, D-dimer, lactate, and albumin, these markers were measured by a real-time manner after blood sampling. CRP was measured with turbidimetric immunoassay (CRPH reagent, Synchron System Beckman, Brea, CA, USA). PCT was measured with enzyme-linked immunosorbent assay (ELISA) (VIDAS BRAHMS PCT, BioMerieux, Durham, England). D-dimer was measured with microlatex immunoturbility assay (Simens Innovance D-Dimer Reagent package insert, Dade Behring, Marburg, Germany). Lactate was measured with enzymatic method (Lactate reagent, Beckman Coulter, Brea, CA, USA). Albumin was measured with colorimetric method (Albumin reagent, KANTO TAIWAN Corporation, Taipei, Taiwan). For evaluating HMGB1, IL-8 and IL-10 and serum and plasma samples were centrifuged at 1000 g for 15 minutes at 4°C within one hour following collection. Samples were immediately stored in 1.5 cc microcentrifuge tube at −80°C until analysis for HMGB1, IL-8, and IL-10. HMGB1, IL-8, and IL-10 were measured with a commercially available ELISA (Quantikine ELISA kit, R&D Systems, Minneapolis, MN, USA), and the frozen samples were preserved within 30 days before ELISA measurement for HMGB1, IL-8, and IL-10.

All clinical factors, serum biomarkers, and the day 3 : day 1 ratios of biomarker concentration or score values were analyzed as potential predictive factors of ICU mortality and 6-month outcomes. Furthermore, the length of the hospital stay, length of ICU stay, and number of mechanical ventilation (MV) days were also recorded as factors to predict 6-month outcomes after hospital discharge.

### 2.4. Statistical Analysis

Categorical variables were analyzed using the Chi-squared test or Fisher's exact test where appropriate, and continuous variables were compared using the Student's *t*-test or the Mann-Whitney *U* test. Multivariate logistic regression analysis was performed to identify independent clinical factors and biomarkers of ICU mortality and 6-month outcome, respectively. All variables considered as risk factors with a *P* value <0.10 by univariate analysis were entered into the multivariate model. If individual variables had a *P* value <0.05 in the multivariate model, a backward elimination procedure was used to identify the final independent risk factors.

Results are presented as absolute numbers (percentage) or mean and standard deviation (SD). Adjusted odds ratios (AORs) and 95% confidence intervals (CIs) were reported for the logistic regression analysis. A 2-tailed *P* value <0.05 was considered significant. All statistical analyses were performed using the Statistical Package for the Social Sciences (SPSS) 14.0 software package (SPSS Inc., Chicago, IL, USA).

## 3. Results

**Figure 1:**
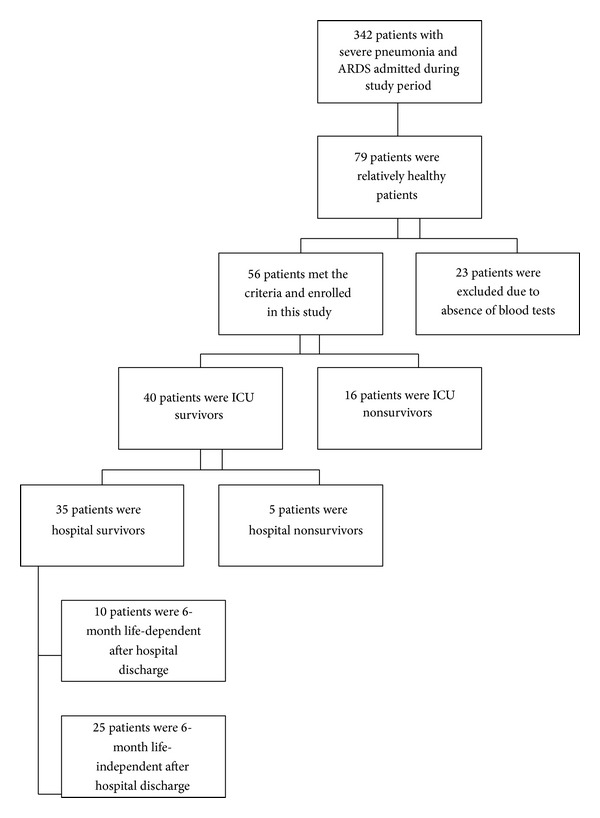
Flow chart of patient selection in this study: 342 patients with severe pneumonia and ARDS were admitted to medical ICUs within the study period, while 56 patients were enrolled in this study. Among the 56 patients, 40 patients were ICU survivors and 16 were ICU nonsurvivors. Only 35 patients were successfully discharged from the hospital, and 25 patients were considered as 6-month life independent.

In total, 342 patients with severe pneumonia and ARDS admitted to medical ICUs within the 2-year period from July 1, 2009, to June 30, 2011, and only 79 relatively healthy patients met the inclusion criteria. Twenty-three patients refused blood tests due to being in critical condition; therefore, only 56 patients were enrolled in our study. Among the 56 patients, 40 patients were ICU survivors and 16 were ICU nonsurvivors, resulting in a mortality rate of 28.6%. Only 35 patients were successfully discharged from the hospital, and 25 patients were considered to be 6-month life independent by Barthel's Index criteria (Figure 1).

### 3.1. Characteristics of ICU Survivors and Nonsurvivors

Table 1 shows the clinical factor characteristics between the ICU survivors and nonsurvivors. We found no statistical intergroup differences in gender, pneumonia type, initial PaO_2_/FiO_2_ value, CCI, and the ratios of the SOFA scores on day 3 to those on day 1. However, we also determined that initial adequate antibiotic use, low physiologic scores for APACHE II, SOFA, and SAPS II at days 1 and 3, and a large day 3 : day 1 value for the APACHE II and SAPS II scores may result in a low ICU mortality rate in patients with severe pneumonia and ARDS.

Table 2 indicates the serum biomarker characteristics between ICU survivors and nonsurvivors. We noted low day 3 levels of CRP and lactate, low day 1 D-dimer level, and low day 1 and day 3 levels of HMGB1 and IL-8 in ICU survivors.

### 3.2. Characteristics of 6-Month Life-Dependent and 6-Month Life-Independent Patients

Table 3 describes the clinical factor characteristics between 6-month life-independent and life-dependent patients. We found that old age, HAP, high CCI value, the ratio of the SOFA scores on day 3 to those on day 1, long ICU or hospital stay, and a high number of MV days may result in 6-month life dependence after hospital discharge.

Table 4 shows the serum biomarker characteristics between 6-month life-independent and life-dependent patients. We determined that low albumin level at day 1 and the day 3 : day 1 ratio for the albumin level, high HMGB1 level at day 1 and day 3, and high lactate level at day 3 may be associated with 6-month life dependence after hospital discharge.

### 3.3. Predictors for ICU Mortality and 6-Month Life Dependence

In this study, we used the multivariate logistic regression method to determine independent clinical factors and biomarkers of predicting ICU mortality and 6-month life dependence, respectively. We found that initial appropriate use of antibiotics (*P* = 0.037) was an independent clinical factor, and HMGB1 concentration at day 1 was an independent biomarker of ICU mortality (Table 5). Using multivariate logistic regression, we found no independent clinical predictor for 6-month life dependence; however, albumin level at day 1 (*P* = 0.049) was confirmed to be an independent biomarker of 6-month life dependence after hospital discharge (Table 6).

## 4. Discussion

Underlying comorbidity has been reported to be an important prognostic factor in critically ill patients and has been shown to be an important determinant of ICU outcome [20–23]. The CCI [24, 25] was developed to predict 1-year mortality among medical patients and is one of the most frequently used measures of comorbidity [26]. Comorbidities are often included in the exclusion criteria while designing trials to avoid their confounding influence on the study outcome. Patients with few underlying diseases tend to have good clinical outcomes even after being in critical condition; however, ICU mortality or long-term disability can result in relatively healthy patients with severe pneumonia and ARDS [13]. A previous study suggested that people with CCI scores >5 have essentially an 84% risk of dying at 1 year [24]; thus, we selected patients with severe pneumonia and ARDS status with CCI scores below 5 to determine the risk factors associated with ICU mortality and 6-month outcomes for clarifying the findings in “relatively healthy patients.”

In this study, we assessed clinical factors such as initial appropriate antibiotic treatment, APACHE II scores, SOFA scores, SAPS II scores on days 1 and 3 after disease onset, and we found that the day 3 : day 1 ratios of the APACHE II and SAPS II scores were associated with ICU mortality. Multivariate analysis revealed that initial appropriate use of antibiotic was an independent clinical factor for ICU mortality. A previous study demonstrated that inappropriate antibiotic treatment for pneumonia was associated with increased mortality [27]. Appropriateness of antibiotic treatment was assessed in terms of antimicrobial coverage, defined as an antimicrobial susceptibility result indicating that the isolated pathogen was sensitive to the administered antibiotic [18]. However, the relevance of pharmacokinetic-pharmacodynamic relationships in optimizing drug exposure, even if the drug is administered in a timely manner, has been progressively highlighted [28, 29]. Timely administration of an optimized dose of the right antibiotic would result in a good clinical response. Regarding this issue, careful assessment of the clinical response would be helpful in determining whether the antibiotic is appropriate. We defined “initial, appropriate use of antibiotic” according to microbiological data as well as the results of an assessment of clinical response. By using such a strict definition, we found that initial appropriate antibiotic treatment was an independent clinical predictor of ICU mortality in relatively healthy patients with severe pneumonia and ARDS.

With regard to serum biomarkers predicting ICU mortality, we found that high serum levels of CRP and the day 3 lactate, day 1 D-dimer, HMGB1, and day 1 or day 3 IL-8 levels were frequently observed in ICU nonsurvivors. Furthermore, day 1 HMGB1 level was an independent biomarker of ICU mortality. Data from some animal studies showed that high HMGB1 levels are associated with severity and lethality of sepsis. HMGB1 has also been reported to be associated with shock [30] and acute lung injury [31], and blocking HMGB1 with an anti-HMGB1 antibody attenuated lung injury in animal studies [32, 33]. In human studies, high HMGB1 level has been associated with increased mortality in subjects with severe sepsis [30]. Another study showed that HMGB1 concentration did not differ between survivors and nonsurvivors and did not predict hospital mortality in patients with severe sepsis [34]. However, these studies did not select a designated patient group such as “relatively healthy patient.” HMGB1 was originally named amphoterin [35] and is secreted by immune cells (e.g., macrophages, monocytes, and dendritic cells) through a leaderless secretory pathway [36]. Activated macrophages and monocytes secrete HMGB1 as a cytokine mediator of inflammation [30]. The immune response in relatively healthy patients would function better than the immune response in patients with a weakened immune system. It is reasonable to anticipate that high HMGB1 concentration may correlate with severity and mortality in relatively healthy patients who have severe pneumonia and ARDS. We also found that day 1 HMGB1 concentration was an independent biomarker of predicting ICU mortality.

With regard to predicting 6-month life dependence, we found that old age, having HAP, high CCI, and prolonged ICU stay, hospital stay, and MV days frequently contributed to 6-month life dependence. Serum biomarkers such as albumin, lactate, and HMGB1 on days 1 or 3 were associated with 6-month life dependence. We also determined that a low day 1 albumin level was an independent biomarker of predicting 6-month life dependence after hospital discharge. Interestingly, a previous report demonstrated that lower serum albumin concentrations could have been caused by ongoing inflammation, poor health status, and malnutrition; these factors have also been shown to be associated with low muscle mass or accelerated muscle loss [37]. Serum albumin concentration is also a marker of underlying diseases that are associated with muscle wasting; a previous study also revealed the association between lower albumin concentration and low muscle mass after careful adjustment for known health factors [37]. Several studies have described the iron-binding antioxidant properties of albumin [38], which is also a specific modulator of cellular glutathione and an important antioxidant [39]. Oxidative damage may play a crucial role in the decline of skeletal muscle that occurs during aging [40]. Taken together, these results and those of our study suggest that albumin may be a marker of skeletal muscle wasting and an independent factor for predicting life dependency in relatively healthy patients with severe pneumonia and ARDS.

The strength of this study was in the patient group selection; patients with severe pneumonia and ARDS that were included in our study had few underlying comorbidities and were relatively healthy patients; this group of patients has seldom been studied previously. In our study, 342 patients were admitted to medical ICUs within the study period and only 79 of these (23.1%) were relatively healthy patients. This also shows the group of relatively healthy patients is rare. By analyzing the data that we collected in our study, we could elucidate possible independent clinical factors and biomarkers of predicting ICU mortality and 6-month life dependence in this patient group.

However, there are still some limitations in our study. First, the study sample size was too small and this might result in a powerless outcome; however the reason for the small sample size may be due to patient characteristics. In other words, few relatively healthy patients with pneumonia would typically progress rapidly to ARDS requiring ICU admission. We tried our best to enroll patients that met our inclusion criteria during the 2-year period, although only 56 patients were able to join our study. Second, not every patient was able to be followed up to 1 year after hospital discharge; while every patient enrolled in this study was followed for a 6-month period, we could not provide 1-year outcome data.

## 5. Conclusions 

Individuals in the general population are usually healthy with little comorbidity and seldom experience of ARDS after pneumonia occurs. However, if pneumonia indeed progresses to ARDS, they will be intubated and require MV and ICU care. This outcome may be unexpected and unfortunate for these relatively healthy patients. Importantly, we determined that initial appropriate antibiotic use (a clinical factor) and the day 1 HMGB1 concentration (a biomarker) were independent factors for predicting ICU mortality, and the day 1 albumin level was an independent biomarker of predicting 6-month life dependence in relatively healthy patients with severe pneumonia and ARDS status.

## Figures and Tables

**Table 1:** Clinical factors characteristics between ICU survivors and nonsurvivors.

	ICU survivors (*n* = 40)	ICU nonsurvivors (*n* = 16)	*P *
Age	63.43 ± 13.95	70.63 ± 9.22	0.063
Sex, male	23	8	0.767
Pneumonia type			0.262
Community acquired	23	7	
Hospital acquired	17	9	
Initial PaO_2_/FiO_2_	163.05 ± 60.68	160.57 ± 58.24	0.889
Charlson's comorbidity index	2.03 ± 1.31	2.62 ± 1.31	0.127
Initial antibiotics			<0.001
Appropriate	17	0	
Inappropriate	8	13	
Indeterminate	15	3	
APACHE II score			
Day 1	25.47 ± 5.29	29.37 ± 5.02	0.015
Day 3	22.45 ± 5.90	30.50 ± 6.59	<0.001
Day 3 : Day 1	0.88 ± 0.11	1.04 ± 0.15	0.001
SOFA score			
Day 1	8.27 ± 2.62	11.38 ± 2.82	<0.001
Day 3	7.53 ± 2.35	11.37 ± 2.63	<0.001
Day 3 : Day 1	0.93 ± 0.21	1.03 ± 0.23	0.147
SAPS II score			
Day 1	41.05 ± 9.12	49.50 ± 11.55	0.005
Day 3	37.70 ± 8.27	50.44 ± 13.39	<0.001
Day 3 : Day 1	0.92 ± 1.01	1.02 ± 0.12	0.003

^a^Continuous variables were analyzed by Student's *t*-test or Mann-Whitney *U* test, and categorical data by Chi-square test.

^b^ICU: intensive care unit; APACHE II: Acute Physiology and Chronic Health Evaluation II; SOFA: Sequential Organ Failure Assessment; and SAPS II: Simplified Acute Physiology Score II.

^c^Variables are expressed as mean (standard deviation) and categorical data are expressed as number (percentage).

**Table 2:** Serum biomarkers characteristics between ICU survivors and nonsurvivors.

	ICU survivors (*n* = 40)	ICU nonsurvivors (*n* = 16)	*P *
CRP (mg/L)			
Day 1	184.68 ± 106.53	179.71 ± 73.53	0.865
Day 3	102.77 ± 69.01	239.66 ± 67.06	<0.001
Day 3 : day 1	0.91 ± 2.01	1.62 ± 1.15	0.184
PCT (ng/mL)			
Day 1	8.29 ± 8.68	9.48 ± 8.32	0.639
Day 3	5.98 ± 11.952	8.37 ± 6.38	0.453
Day 3 : day 1	2.67 ± 9.38	1.05 ± 0.48	0.496
D-dimer (mg/L)			
Day 1	4.08 ± 2.07	5.58 ± 2.87	0.034
Day 3	4.07 ± 4.12	5.56 ± 2.45	0.167
Day 3 : day 1	1.28 ± 2.13	1.31 ± 1.23	0.945
Lactate (mg/dL)			
Day 1	22.21 ± 9.67	5.58 ± 2.87	0.235
Day 3	16.03 ± 7.69	32.34 ± 27.80	0.001
Day 3 : day 1	0.83 ± 0.50	1.86 ± 3.38	0.061
Albumin (g/dL)			
Day 1	2.56 ± 0.49	2.45 ± 0.39	0.416
Day 3	2.59 ± 0.47	2.36 ± 0.35	0.080
Day 3 : day 1	1.02 ± 0.13	0.97 ± 0.11	0.195
HMGB1 (pg/mL)			
Day 1	1614.14 ± 641.11	2108.51 ± 574.51	0.025
Day 3	1548.81 ± 650.96	1952.45 ± 763.77	0.010
Day 3 : Day 1	1.02 ± 0.39	1.00 ± 0.52	0.908
IL-8 (pg/mL)			
Day 1	56.24 ± 38.10	173.18 ± 304.77	0.019
Day 3	48.27 ± 22.45	97.19 ± 44.29	<0.001
Day 3 : Day 1	1.12 ± 0.79	0.97 ± 0.42	0.472
IL-10 (pg/mL)			
Day 1	40.01 ± 35.10	49.08 ± 66.91	0.509
Day 3	39.15 ± 35.56	39.27 ± 28.05	0.990
Day 3 : Day 1	1.16 ± 0.83	1.28 ± 0.63	0.616

^a^Continuous variables were analyzed by Student's *t*-test or Mann-Whitney *U* test, and categorical data by Chi-square test.

^b^ICU: intensive care unit; CRP: c-reactive protein; PCT: procalcitonin; HMGB1: high-mobility group protein B1; and IL: interleukin.

^c^Variables are expressed as mean (standard deviation) and categorical data are expressed as number (percentage).

**Table 3:** Clinical characteristics of 6-month life-independent and life-dependent patients.

	6-month life independence (*n* = 25)	6-month life dependence (*n* = 10)	*P *
Age	57.12 ± 12.51	75.30 ± 8.07	<0.001
Sex, male	16	5	0.474
Pneumonia type			0.002
Community acquired	20	2	
Hospital acquired	5	8	
Initial PaO_2_/FiO_2_	173.13 ± 63.84	135.76 ± 56.83	0.117
Charlson's Comorbidity Index	1.64 ± 1.15	3.20 ± 1.13	0.001
Initial antibiotics			1.000
Appropriate	10	4	
Inappropriate	5	2	
Indeterminate	10	4	
APACHE II score			
Day 1	24.72 ± 5.22	25.80 ± 1.64	0.584
Day 3	21.04 ± 4.63	23.30 ± 5.37	0.221
Day 3 : Day 1	0.85 ± 0.11	0.91 ± 0.11	0.209
SOFA score			
Day 1	8.00 ± 2.75	8.20 ± 1.69	0.833
Day 3	7.52 ± 2.42	6.80 ± 2.10	0.416
Day 3 : Day 1	0.96 ± 0.20	0.82 ± 0.13	0.044
SAPS II score			
Day 1	39.24 ± 7.17	43.90 ± 12.92	0.181
Day 3	36.36 ± 7.92	39.60 ± 10.29	0.323
Day 3 : Day 1	0.93 ± 0.09	0.91 ± 0.09	0.759
ICU stay	17.68 ± 8.57	27.00 ± 8.56	0.007
Hospital stay	32.80 ± 16.79	61.20 ± 19.20	<0.001
MV day	15.24 ± 8.04	23.30 ± 7.62	0.010

^a^Continuous variables were analyzed by Student's *t*-test or Mann-Whitney *U* test, and categorical data by Chi-square test.

^b^ICU: intensive care unit; APACHE II: Acute Physiology and Chronic Health Evaluation II; SOFA: Sequential Organ Failure Assessment; SAPS II: Simplified Acute Physiology Score II; MV: mechanical ventilation.

^c^Variables are expressed as mean (standard deviation) and categorical data are expressed as number (percentage).

**Table 4:** Serum biomarker characteristics between 6-month life-independent and life-dependent patients.

	6-month life independence (*n* = 25)	6-month life dependence (*n* = 10)	*P *
CRP (mg/L)			
Day 1	174.40 ± 93.51	205.24 ± 151.19	0.468
Day 3	98.52 ± 70.53	97.91 ± 78.28	0.982
Day 3 : day 1	1.09 ± 2.53	0.51 ± 0.20	0.480
PCT (ng/mL)			
Day 1	8.15 ± 9.47	8.41 ± 7.40	0.939
Day 3	3.66 ± 3.34	11.32 ± 22.53	0.101
Day 3 : day 1	3.35 ± 11.57	1.97 ± 4.44	0.717
D-dimer (mg/L)			
Day 1	4.12 ± 2.18	4.06 ± 1.19	0.928
Day 3	4.57 ± 4.99	3.06 ± 1.68	0.358
Day 3 : day 1	1.52 ± 2.67	0.78 ± 0.38	0.390
Lactate (mg/dL)			
Day 1	20.01 ± 8.37	26.32 ± 13.06	0.090
Day 3	13.40 ± 7.16	19.45 ± 7.84	0.035
Day 3 : Day 1	0.80 ± 0.60	0.84 ± 0.36	0.849
Albumin (g/dL)			
Day 1	2.76 ± 0.39	2.18 ± 0.49	0.001
Day 3	2.73 ± 0.47	2.39 ± 0.39	0.051
Day 3 : Day 1	0.99 ± 0.13	1.11 ± 0.15	0.017
HMGB1 (pg/mL)			
Day 1	1425.36 ± 578.39	1881.06 ± 731.29	0.049
Day 3	1281.25 ± 492.45	2009.14 ± 754.99	0.002
Day 3 : Day 1	0.95 ± 0.34	1.20 ± 0.50	0.103
IL-8 (pg/mL)			
Day 1	55.52 ± 33.40	49.27 ± 53.66	0.678
Day 3	50.80 ± 23.57'	35.63 ± 7.95	0.057
Day 3 : Day 1	1.07 ± 0.58	1.38 ± 1.28	0.317
IL-10 (pg/mL)			
Day 1	39.72 ± 32.64	34.88 ± 42.36	0.719
Day 3	43.03 ± 36.08	25.35 ± 26.73	0.171
Day 3 : Day 1	1.18 ± 0.88	1.20 ± 0.82	0.941

^a^Continuous variables were analyzed by Student's *t*-test or Mann-Whitney *U* test, and categorical data by Chi-square test.

^b^CRP: C-reactive protein; PCT: procalcitonin; HMGB1: high-mobility group protein B1; and IL: interleukin.

^c^Variables are expressed as mean (standard deviation) and categorical data are expressed as number (percentage).

**Table 5:** Multivariate logistic regression analysis for identification of independent clinical factors and biomarkers of predicting ICU mortality.

Variable	Adjusted odds ratio	95% Confidence interval	*P *
Clinical factors for predicting ICU mortality			
Age	1.506	0.807–2.810	0.198
Initial appropriate antibiotics use	0.032	0.001–0.814	0.037
APACHE II score			
Day 1	0.001	<0.001–90.210	0.239
Day 3	1024.53	0.009–1.187	0.244
Day 3 : day 1	8.566	0.003–27664.475	0.602
SOFA score			
Day 1	0.032	<0.001–34.761	0.335
Day 3	2.756	0.711–10.687	0.143
SAPS II score			
Day 1	2.222	0.111–44.423	0.601
Day 3	0.558	0.030–10.234	0.694
Day 3 : Day 1	0.469	<0.001–28252.867	0.893

Biomarkers for predicting ICU mortality			
CRP (mg/L) day 3	1.265	0.798–2.005	0.317
D-dimer (ng/mL) day 1	1.211	0.818–1.793	0.339
Lactate (mg/dL) ratio	0.879	0.005–163.371	0.879
Albumin (g/dL) day 3	1.075	<0.001–7.976	0.261
HMGB1 (pg/mL)			
Day 1	1.002	1.000–1.004	0.026
Day 3	0.990	0.968–1.013	0.384
IL8 (pg/mL)			
Day 1	1.039	0.955–1.130	0.737
Day 3	1.075	0.940–1.229	0.291

^a^ICU: intensive care unit; APACHE II: Acute Physiology and Chronic Health Evaluation II; SOFA: Sequential Organ Failure Assessment; SAPS II: Simplified Acute Physiology Score II; CRP: C-reactive protein; HMGB1: high-mobility group protein B1; and IL: interleukin.

**Table 6:** Multivariate logistic regression analysis for identification of independent clinical factors and biomarkers of predicting 6-month life dependence.

Variables	Adjusted odds ratio	95% Confidence interval	*P *
Clinical factors for predicting 6-month life dependence			
Age	0.500	0.196~1.277	0.147
Pneumonia type	0.067	0.001~3.803	0.190
Charlson's comorbidity index	0.046	0.001~1.827	0.101
ICU stay	0.083	0.001~5.067	0.235
Hospital stay	0.861	0.670~1.105	0.240
MV day	1.134	0.851~1.512	0.390

Biomarkers of predicting 6-month life dependence			
Lactate (mg/dL)			
Day 1	0.909	0.737~1.127	0.385
Day 3	0.944	0.808~1.102	0.465
Albumin (g/dL)			
Day 1	18.675	1.018~342.677	0.049
Day 3	0.415	0.016~10.551	0.595
Day 3 : Day 1	0.056	0.000~148.527	0.474
HMGB1 (pg/mL)			
Day 1	1.003	0.999~1.006	0.099
Day 3	0.994	0.989~1.000	0.068

^a^ICU: intensive care unit; MV: mechanical ventilation; HMGB1: high-mobility group protein B1.

## Abbreviations

ALI: Acute lung injury
AORs: Adjusted odds ratios
APACHE II: Acute Physiology and Chronic Health Evaluation II
ARDS: Acute respiratory distress syndrome
CAP: Community-acquired pneumonia
CCI: Charlson's comorbidity index
CIs: Confidence intervals
CRP: C-reactive protein levels
ELISA: Enzyme-linked immunosorbent assay
HAP: Hospital-acquired pneumonia
HMGB1: High-mobility group protein B1
ICU: Intensive care unit
IL: Interleukin
MV: Mechanical ventilation
PaO_2_/FiO_2_: Arterial oxygen tension/fraction inspired oxygen
PCT: Procalcitonin
QOL: Quality of life
SAPS: Simplified Acute Physiology Score
SD: Standard deviation
SOFA: Sequential Organ Failure Assessment.
